# Ectopic Leptin Production by Intraocular Pancreatic Islet Organoids Ameliorates the Metabolic Phenotype of *ob/ob* Mice

**DOI:** 10.3390/metabo11060387

**Published:** 2021-06-14

**Authors:** Barbara Leibiger, Tilo Moede, Ismael Valladolid-Acebes, Meike Paschen, Montse Visa, Ingo B. Leibiger, Per-Olof Berggren

**Affiliations:** The Rolf Luft Research Center for Diabetes and Endocrinology, Department of Molecular Medicine and Surgery, Karolinska Institutet, Karolinska Sjukhuset L1:03, 17176 Stockholm, Sweden; tilo.moede@ki.se (T.M.); ismael.valladolid.acebes@ki.se (I.V.-A.); paschen.meike@gmail.com (M.P.); montserrat.visa.majoral@ki.se (M.V.); ingo.leibiger@ki.se (I.B.L.)

**Keywords:** pancreatic islets, leptin, exocytosis, tissue-engineering, in vivo imaging, metabolism, diabetes, β-cell, viral transduction, transplantation

## Abstract

The pancreatic islets of Langerhans consist of endocrine cells that secrete peptide hormones into the blood circulation in response to metabolic stimuli. When transplanted into the anterior chamber of the eye (ACE), pancreatic islets engraft and maintain morphological features of native islets as well as islet-specific vascularization and innervation patterns. In sufficient amounts, intraocular islets are able to maintain glucose homeostasis in diabetic mice. Islet organoids (pseudo-islets), which are formed by self-reassembly of islet cells following disaggregation and genetic manipulation, behave similarly to native islets. Here, we tested the hypothesis that genetically engineered intraocular islet organoids can serve as production sites for leptin. To test this hypothesis, we chose the leptin-deficient *ob/ob* mouse as a model system, which becomes severely obese, hyperinsulinemic, hyperglycemic, and insulin resistant. We generated a Tet-OFF-based beta-cell-specific adenoviral expression construct for mouse leptin, which allowed efficient transduction of native beta-cells, optical monitoring of leptin expression by co-expressed fluorescent proteins, and the possibility to switch-off leptin expression by treatment with doxycycline. Intraocular transplantation of islet organoids formed from transduced islet cells, which lack functional leptin receptors, to *ob/ob* mice allowed optical monitoring of leptin expression and ameliorated their metabolic phenotype by improving bodyweight, glucose tolerance, serum insulin, and C-peptide levels.

## 1. Introduction

Pancreatic islets of Langerhans are micro-organs that form the endocrine part of the pancreas. They consist of endocrine alpha-, beta-, delta-, epsilon- and PP-cells, that produce and secrete glucagon, insulin, somatostatin, ghrelin, and pancreatic polypeptide, respectively. The fenestrated islet blood vessels allow for an efficient exchange of blood-derived factors stimulating these cells as well as the immediate release of the secreted hormones into the blood circulation. Importantly, these characteristics are maintained when pancreatic islets are transplanted into the anterior chamber of the eye, ACE [1]. In fact, transplantation of a sufficient number of islets to the ACE (75–300 islets in mice) showed that these islet grafts are capable of maintaining glycaemia in streptozotocin-treated diabetic mice ([1], reviewed in [2]). Islet organoids (also called pseudo-islets) that are formed by self-reassembly of islet cells following disaggregation and genetic manipulation, for example by adenoviral-mediated ectopic gene expression [3,4], behave similarly to native islets. Because of these features, we wanted to test the hypothesis that genetically engineered intraocular islet organoids can serve as production sites for blood-born proteins/peptides as a novel treatment strategy. To test this hypothesis, we chose the *ob/ob* mouse as a disease model. *Ob/ob* mice lack functional leptin, which is an adipocyte-produced peptide hormone, and develop a very pronounced phenotype based, among others, on their extreme hyperphagia. After weaning, they become severely obese, hyperinsulinemic, hyperglycemic, and insulin resistant (reviewed in [5]). Treating these mice with peripherally administered leptin reverses [6,7] or ameliorates this phenotype in a dose-dependent manner [8]. Ectopic expression of leptin via gene therapy approaches by either injecting virus-encoded leptin constructs [9,10] or transplanting engineered leptin-expressing cells [11,12] improves the metabolic phenotype of *ob/ob* mice even further. Because leptin acts in concert with insulin as anorexic stimuli in the central nervous system (reviewed in [13]), we decided to genetically engineer insulin-producing beta-cells to become production sites for leptin. This allowed us to test the hypothesis whether ectopic leptin production by islet organoids in the ACE ameliorates the metabolic phenotype of *ob/ob* mice.

## 2. Results

### 2.1. Generation of a Tet-OFF-Based Beta-Cell Specific Adenoviral Expression Construct for Mouse Leptin and Its In Vitro Assessment

**Figure 1:**
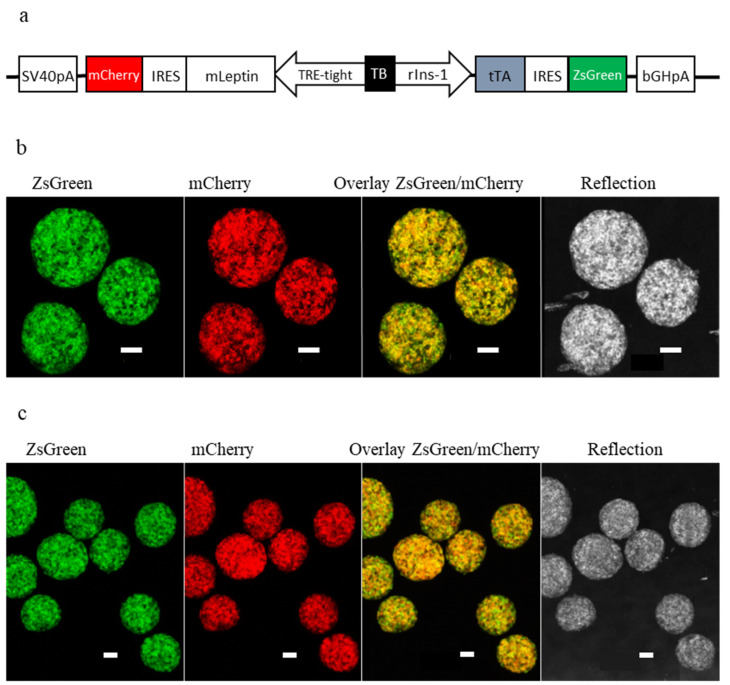
In vitro characterization of leptin-expressing islet organoids. (**a**) Schematic illustration of vAd-RIP-leptin-OFF. The rat insulin-1 promoter (rIns-1) drives expression of the synthetic transcription factor tTA and the green fluorescent protein ZsGreen in pancreatic beta-cells. The TRE-tight promoter drives expression of mouse leptin (mLeptin) and the red fluorescent protein mCherry. The two expression cassettes are separated by a transcription blocker sequence (TB). Binding of tTA to the TRE-tight promoter induces in the absence of doxycycline the expression of leptin and mCherry, while addition of doxycycline turns-off the expression of the two proteins. IRES-elements in the cassettes ensures stoichiometric expression of the two proteins under the same promoter. (**b**) Representative maximum projection of a 3D-stack of leptin-expressing islet organoids, obtained by confocal imaging, cultured for 4 weeks in vitro (1st set of experiments), showing reflection, expression of ZsGreen, mCherry and their overlay. Scale bar: 50 µm. (**c**) Representative maximum projection of a 3D-stack of leptin-expressing islet organoids, obtained by confocal imaging, cultured for 4 weeks in vitro (2nd set of experiments), showing reflection, expression of ZsGreen, mCherry and their overlay. Scale bar: 50 µm.

When designing the expression construct for mouse leptin, we considered the following points: We wanted (1) leptin to be expressed in pancreatic beta-cells, (2) to be able to reduce/turn-off leptin expression if necessary, (3) to optically monitor the activity of the expression construct by fluorescence microscopy, and (4) an adenoviral-based expression construct for efficient transduction of native beta-cells. As a result, we generated an adenoviral vector, vAd-RIP-leptin-OFF, that contains two expression cassettes that are positioned in opposite directions and are separated by a ‘transcription-block’ sequence to allow independent expression (Figure 1a). The first expression cassette allows rat insulin-1 promoter-driven expression of the synthetic transcription factor tTA (Tet-OFF) and the green fluorescent protein ZsGreen in pancreatic beta-cells. The second expression cassette consists of a TRE-tight promoter-driven mouse leptin-IRES-mCherry cassette. Binding of tTA to the TRE-tight promoter induces the expression of leptin and the red fluorescent protein mCherry in beta-cells. Addition of doxycycline inhibits binding of tTA and turns-off the expression of the two proteins. The IRES-element in the two expression cassettes allows the co-expression of tTA with ZsGreen and leptin with mCherry. Hence, ‘green’ and ‘red’ serve as visual read-outs for the expression of tTA and leptin in beta-cells, respectively. For in vitro assessment of the expression construct, we used organoids created from transduced islets cells of B6.BKS(D)-Lepr^db^/J (*db/db*) mice. Organoids from the same preparations were also used for the transplantations. The detection of green and red fluorescence by laser-scanning confocal microscopy in beta-cells of the islet organoids verified that both expression cassettes were active in the same cells (Figure 1b,c). The islet organoids secreted leptin into the culture medium, the secretion was glucose-dependent: 7.02 ± 1.76 pg/organoid/h at 3 mM glucose vs. 12.94 ± 2.83 pg/organoid/h at 16 mM glucose (stimulation index 16 versus 3 mM glucose: 1.93 ± 0.14).

### 2.2. Ectopic Leptin Production by Islet Organoid Grafts in the ACE Ameliorates the Metabolic Phenotype of ob/ob Mice

To test whether intraocular leptin production by islet organoids affects the metabolic phenotype of *ob/ob* mice, we considered the following points. Because leptin deficiency after weaning leads to a rapid development of the *ob/ob* phenotype, including strong beta-cell proliferation in both the native in situ islets as well as in islets transplanted to the ACE and leptin treatment decelerates this process [14], we decided to treat *ob/ob* mice with daily intraperitoneal injections of leptin (1.5 µg/g bodyweight/day) immediately after their arrival from the vendor until 4 weeks after transplantation of the islet organoid grafts, i.e., until their full engraftment. Moreover, because leptin is discussed in the literature to have a negative effect on insulin secretion [15], we decided to avoid the potential negative feed-back of beta-cell produced leptin on the organoids by generating them from islets of leptin receptor-deficient mice, i.e., islets from *db/db* mice with a B6 genetic background (B6.BKS(D)-Lepr^db^/J) to match the B6 background of the *ob/ob* recipient mice (B6.Cg-Lep^ob^/J). Hence, we isolated islets from *db/db* mice, disaggregated them, transduced the islets cells with vAd-RIP-leptin-OFF and generated islet organoids by self-reassembly.

**Figure 2:**
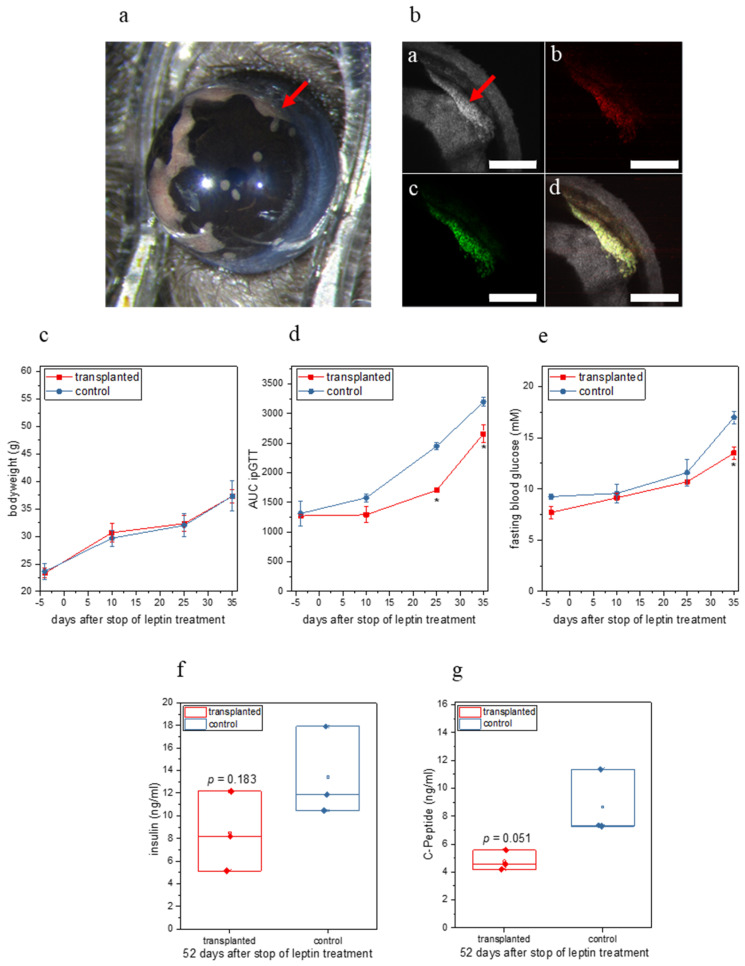
In vivo characterization of leptin-expressing islet organoids from the first set of experiments. (**a**) Photograph of the eye containing the leptin-expressing islet organoid graft (red arrow) 9 weeks after transplantation. (**b**) Maximum projection of a 3D-stack of a leptin-expressing islet organoid graft (red arrow) obtained by confocal imaging 9 weeks after transplantation; (**ba**) reflection, (**bb**) mCherry fluorescence, (**bc**) ZsGreen fluorescence, (**bd**) overlay image; scale bar 500 µm. (**c**) Bodyweight of transplanted and control mice over the entire period of the experiment. (**d**) Area under the curve (AUC) for ipGTT of transplanted and control mice over the entire period of the experiment. (**e**) Fasting blood glucose of transplanted and control mice over the entire period of the experiment. (**f**) Plasma insulin levels of transplanted and control mice at the end of the experiment. (**g**) Plasma C-peptide levels of transplanted and control mice at the end of the experiment. (**d**,**e**) * *p* < 0.05. (**c**–**g**) *n =* 3.

**Figure A1:**
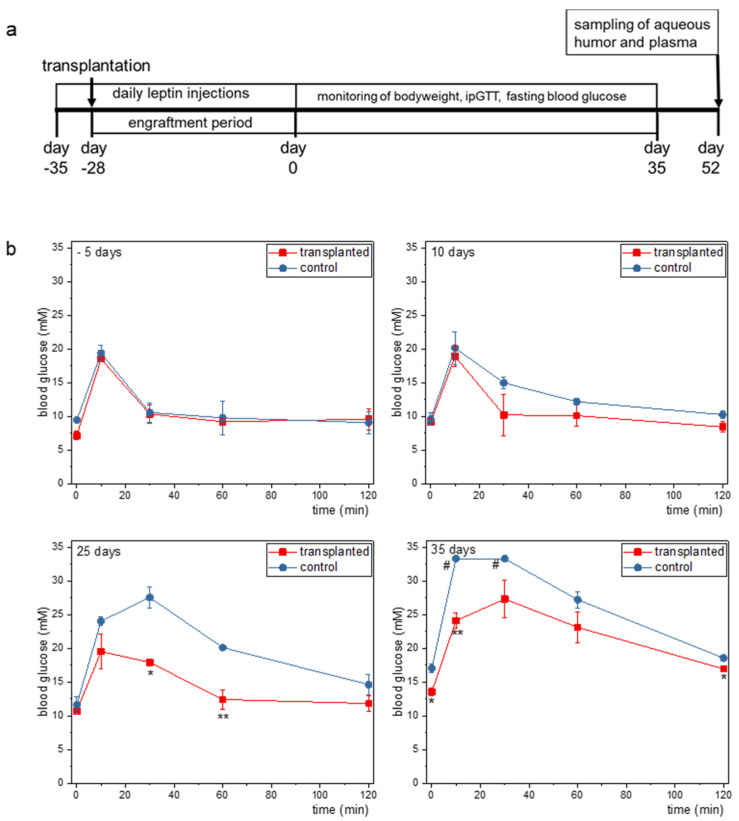
Additional information related to Figure 2. (**a**) Timeline of the first set of experiments. (**b**) Individual traces from the ipGTTs summarized as AUC in Figure 2d. Data are expressed as mean ± SEM. Data points that include saturated glucose measurements that were set to 33.3 mM are labeled with #. * *p* < 0.05; ** *p* < 0.01; *n* = 3.

In a first set of experiments (Figure A1a) we transplanted 130 leptin-expressing *db/db* islet organoids into the ACE of female *ob/ob* mice (*n =* 3, see Figure 2a,b) that were treated with leptin from 15 d before transplantation until 28 d after transplantation and used leptin-treated (for the same time period) female *ob/ob* mice that were not transplanted with islet organoids as a control group (*n =* 3). To use non-transplanted leptin-treated *ob/ob* mice as controls was based on previous observations showing extreme growth of non-leptin expressing intraocular islet grafts in *ob/ob* mice, which required premature termination of experiments for ethical reasons. Inspection of islet organoids kept in vitro (Figure 1b) as well as organoids transplanted to the ACE in vivo (Figure 2b) showed expression of both green and red fluorescent proteins, indicating that both the tTA-IRES-ZsGreen and mLeptin-IRES-mCherry expression cassettes were expressed in beta-cells. While we did not observe a difference in the bodyweight of mice between the two groups (Figure 2c), we observed an improvement in glucose tolerance starting 10 d after stopping leptin treatment, which became significant 25 d after stopping leptin treatment in the transplanted group (Figure 2d and Figure A1b).

Moreover, in the group transplanted with leptin-producing organoids we observed a trend towards a decrease in fasting blood glucose (Figure 2e), which was significant at 35 d after stop of leptin treatment, a trend towards lower plasma insulin (Figure 2f) and insulin C-peptide (Figure 2g) levels, both measured at the end of the experiment. Finally, we detected leptin in the aqueous humor (11.375 ± 3.211 ng/mL) at the end of the experiment in the transplanted group, while no leptin was detectable in the control group. No leptin was detectable in the blood plasma from both groups, as the levels in the transplanted group were likely below the detection limit of the ELISA kit used.

**Figure 3:**
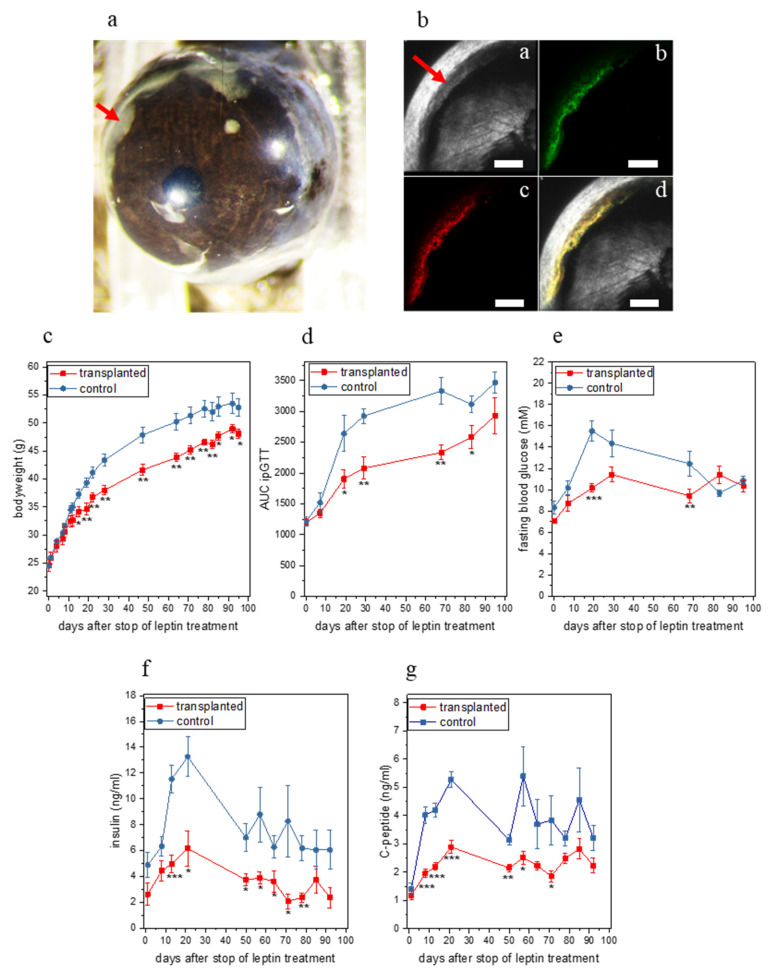
In vivo characterization of leptin-expressing islet organoids from the second set of experiments. (**a**) Photograph of the eye containing the leptin-expressing islet organoid graft (red arrow) 6 weeks after transplantation. (**b**) Maximum projection of a 3D-stack of a leptin-expressing islet organoid graft (red arrow) obtained by confocal imaging 12 weeks after transplantation; (**ba**) reflection, (**bb**) mCherry fluorescence, (**bc**) ZsGreen fluorescence, (**bd**) overlay image; scale bar 300 µm. (**c**) Bodyweight, (**d**) Area under the curve (AUC) for ipGTT, (**e**) Fasting blood glucose, (**f**) Plasma insulin levels and (**g**) Plasma C-peptide levels of transplanted and control mice over the period of the experiment. (**c**–**g**) * *p* < 0.05, ** *p* < 0.01, *** *p* < 0.001; *n =* 7 for transplanted and 5 for control group.

**Figure A2:**
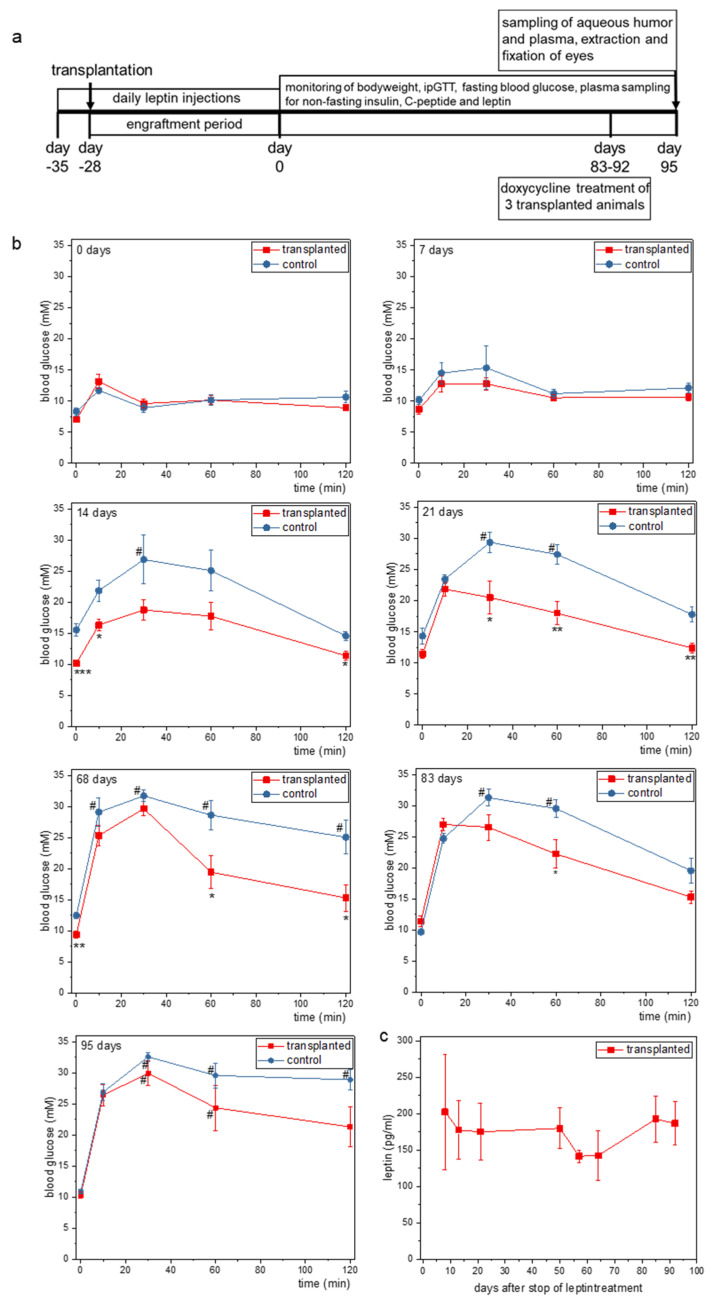
Additional information related to Figure 3. (**a**) Timeline of the second set of experiments. (**b**) Individual traces from the ipGTTs summarized as AUC in Figure 3d. Data are expressed as mean ± SEM. Data points that include saturated glucose measurements are labeled with #. * *p* < 0.05; ** *p* < 0.01; *** *p* < 0.001; *n =* 7 for transplanted and 5 for control group. (**c**) Plasma leptin levels of transplanted mice over the period of the experiment, *n* = 3–7.

In a second set of experiments (Figure A2a) we transplanted 200 leptin-expressing *db/db* islet organoids into the ACE of female *ob/ob* mice that were treated with leptin from 7 d before until 28 d after transplantation (*n =* 7, Figure 3a,b) and used leptin-treated female *ob/ob* mice that were not transplanted with islet organoids as a control group (*n =* 5).

Inspection of islet organoids kept in vitro (Figure 1c) as well organoids transplanted to the ACE in vivo (Figure 3b) showed expression of both green and red fluorescent proteins, indicating that both the tTA-IRES-ZsGreen and mLeptin-IRES-mCherry expression cassettes were expressed in beta-cells. Importantly in this experiment, mice that were transplanted with leptin-producing organoids showed a significant difference in bodyweight from 15 d after stop of leptin treatment (Figure 3c). We observed a significant improvement in ipGTT starting from 19 d after stop of leptin treatment (Figure 3d and Figure A2b). Moreover, we observed a decrease in fasting blood glucose (Figure 3e, significant on days 19 and 68 after stop of leptin treatment), a significant decrease in plasma insulin (from day 14 after stop of leptin, Figure 3f) and a significant decrease in plasma C-peptide levels (from day 8 after stop of leptin, Figure 3g) in the group transplanted with leptin-producing organoids. Finally, we detected leptin in the aqueous humor (6.05 ± 2.91 ng/mL) of the transplanted group at the end of the experiment while no leptin was detectable in the control group. The values of leptin in plasma of the transplanted group were 185.52 ± 10.73 pg/mL between day 8 after stop of leptin treatment and the end of the experiment (Figure A2c). No leptin was detectable in plasma from the control group.

### 2.3. Doxycycline Treatment Stops Ectopic Leptin Expression

**Figure 4:**
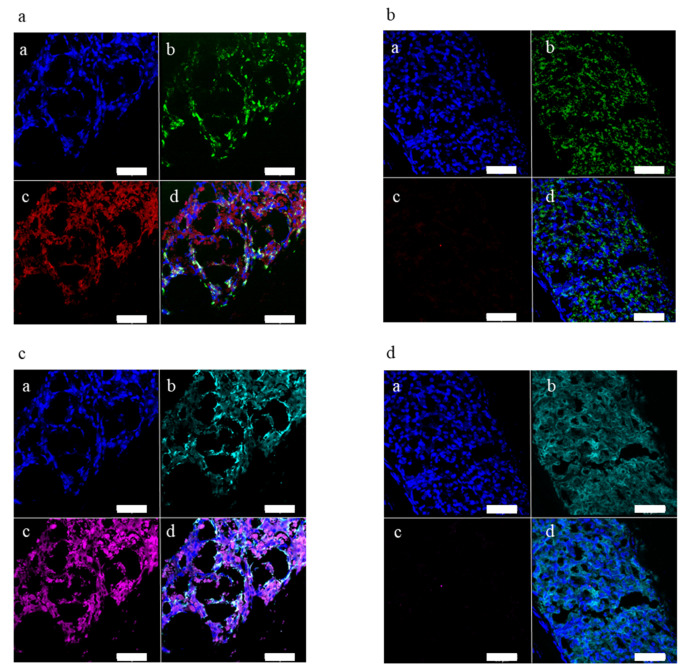
Characterization of leptin-expressing islet organoid grafts from animals treated with doxycycline, removed at the end of the experiment. (**a**) Fluorescent microscopy image of a leptin-expressing islet organoid graft from transplanted non-doxycycline-treated animal; (**aa**) DAPI staining; (**ab**) ZsGreen fluorescence; (**ac**) mCherry fluorescence; (**ad**) overlay. (**b**) Fluorescent microscopy image of a leptin-expressing islet organoid graft from transplanted doxycycline-treated animal; (**ba**) DAPI staining, (**bb**) ZsGreen fluorescence, (**bc**) mCherry fluorescence, (**bd**) overlay. (**c**) Immunohistochemistry image of a leptin-expressing islet organoid graft from transplanted non-doxycycline treated animal; (**ca**) DAPI staining, (**cb**) C-peptide staining, (**cc**) leptin staining, (**cd**) overlay. (**d**) Immunohistochemistry image of a leptin-expressing islet organoid graft from transplanted doxycycline treated animal; (**da**) DAPI staining, (**db**) C-peptide staining, (**dc**) leptin staining, (**dd**) overlay. (**a**–**d**) All scale bars 50 µm.

Three animals of the transplanted group were treated with doxycycline (five intraperitoneal injections of 50 µg dox/kg/mouse over 10 days) to switch-off leptin production from the transplanted organoids. In grafts of these animals no mCherry fluorescence was detectable (Figure 4(bc)) and no leptin expression could be detected by immunohistochemistry (Figure 4(dc)) at the end of the experiment. Expression of only green (Figure 4(bb)), but not red (Figure 4(bc)), fluorescent protein in the graft of doxycycline-treated animals indicates that only the tTA-IRES-ZsGreen cassette was expressed in beta-cells and the expression of the mLeptin-IRES-mCherry cassette was switched-off. In grafts of non-doxycycline treated animals both mCherry and leptin expression were detectable (Figure 4(ac,cc)). More importantly, no leptin was measurable in the aqueous humor of doxycycline-treated animals (*n =* 3).

## 3. Discussion

We have previously shown that the ACE as a transplantation site allows (i) optical/microscopic monitoring of graft vascularization and function), (ii) monitoring of potential pharmacological intervention (systemic or ocular/intraocular treatment), and (iii) the possibility to remove the graft if needed (e.g., by iridectomy). Moreover, hormones secreted by intraocular pancreatic islets are capable of maintaining glucose homeostasis in a diabetic recipient (reviewed in [2]). In this proof-of-concept study we aimed to test whether genetically engineered islet organoids can serve as production sites for non-islet blood-born peptides/proteins, here leptin, and take advantage of the above-listed properties of the ACE as a transplantation site. We designed an adenoviral vector for beta-cell specific expression of mouse leptin that in addition allowed to monitor the activity of the expression cassettes by fluorescence microscopy and to switch-off leptin expression by doxycycline treatment. Expression of leptin in beta-cells brought the potential advantage of glucose-regulated leptin secretion. In addition to the glucose-regulated route of secretion, pancreatic beta-cells are also equipped for continuous release of proteins [16]. We used leptin-expressing islet organoids generated from *db/db* mouse pancreatic islets. Here, all islet cells lack the expression of the functional/signaling leptin receptor and thus do not allow a potential feedback action of secreted leptin on the organoid graft. We chose *ob/ob* mice as recipients, because these mice lack expression of functional leptin and thus develop a severe metabolic phenotype that is easy to monitor, i.e., increased bodyweight, hyperglycemia, and hyperinsulinemia. By transplanting leptin-expressing islet organoids to the ACE we could show that leptin, secreted by the organoids, ameliorates the metabolic phenotype of the *ob/ob* mice. Transplanted mice showed significantly reduced bodyweight, improved glucose tolerance, decreased fasting blood glucose and significant decreased plasma insulin and C-peptide levels. The food intake of these animals was not measured because it would require single animal housing to get reliable results, which was not possible due to the limited number of experimental animals and limitations set by our ethical permits. IpITTs could not be performed due to restrictions of our ethical permits not allowing us to fast the animals more than two times per month. Ectopically expressed leptin was detectable in the aqueous humor and plasma of transplanted mice, whereas no leptin was detected in non-transplanted mice. It is noteworthy that despite the additional beta-cell volume due to the islet organoid grafts, we observed a decrease in serum insulin and C-peptide levels as a result of the ectopic leptin expression. When some of the animals in our study were treated with doxycycline, the co-expression of leptin and mCherry was stopped, demonstrating that the ectopic leptin expression can be switched-off in vivo by the Tet-OFF part of the adenoviral construct.

Our results are comparable to those obtained from *ob/ob* mice that were transplanted with engineered and encapsulated gut-derived K-cells to the peritoneal cavity [11] or with engineered leptin-secreting skin grafts [12]. Additionally, leptin, produced by encapsulated engineered adipocytes and transplanted into visceral fat depots of *ob/ob* mice, was shown to reduce bodyweight and improve glucose tolerance [17].

Although we achieved long-term action of leptin, secreted from the transplanted islet organoids, the effect on glucose metabolism started to fade by the end of the experiment. This can be due to multiple reasons including among others immune response to adenoviral-transduced cells or the transgene [9,18], leading to development of anti-leptin antibodies [9] and increased demand for insulin from transplanted islet organoids, which can lead to hypertrophy and cell proliferation, thereby “diluting out” the leptin-producing cells [6,19]. The limited longevity of adenovirus-mediated expression has been discussed in the past [20,21].

Our study with relative low numbers of animals shows encouraging results. Future work is needed to confirm our preliminary conclusions. In the future, it will be possible to generate islet organoids from genetically engineered stem-cell derived material. This will not only help to overcome the shortage of supply of primary pancreatic islets but will allow engineering the cellular components for an optimized protein production site.

In summary, we have demonstrated that ectopic leptin production by genetic engineered islet organoids, transplanted into the ACE, ameliorates the obese and diabetic phenotype in leptin-deficient *ob/ob* mice. Using the ACE as the transplantation site allowed for optical monitoring of ectopic gene expression by the use of co-expressed fluorescent proteins and gives the safety potential to remove the intraocular graft by for example iridectomy.

In conclusion, our data provide a proof-of-concept that genetically engineered islet organoids can serve, as entire micro-organs, as factories for non-islet blood-born peptides in high enough concentrations to have a physiological function, here exemplified by the production of leptin.

## 4. Materials and Methods

### 4.1. Animals

Female B6.Cg-Lep^ob^/J (*ob/ob*) mice at 4 weeks of age and B6.BKS(D)-Lepr^db^/J (*db/db*) mice at 8 weeks of age were purchased from Jackson Laboratories (Bar Harbour, ME, USA). Both mouse strains share a C57BL/6J (B6) background. After delivery, the mice were allowed to adapt to the animal facility for 1 week before the start of the experiment. All mice were group-housed on a 12/12-h dark/light cycle with free access to food (chow diet R70 from Lantmännen, Kimstad, Sweden) and water. All experiments were performed in accordance with the Karolinska Institutet’s guidelines for the care and use of animals in research and were approved by the institute’s Animal Ethics Committee.

### 4.2. Leptin Treatment of ob/ob Mice

*Ob/ob* mice were treated daily with one intraperitoneal injection of recombinant human leptin protein (1.5 µg/g bodyweight, R&D Systems, Minneapolis, MN, USA) from the day of arrival until 4 weeks after transplantation.

### 4.3. Expression Vector Construction

The mouse leptin cDNA was obtained from pCMV6.mouse leptin (#MC208876, OriGene, Rockville, MD, USA) and subcloned into pTRE-tight (Clontech, Takara, Mountain View, CA, USA) to create pTRE-tight.mLeptin. Next, we inserted an IRES-mCherry sequence downstream of the mLeptin cDNA to obtain pTRE-tight.mLeptin-IRES-mCherry. To create pENTR.TRE-tight.mLeptin-IRES-mCherry/RIP1.DsRed2 we exchanged in pENTR.rbGK.EGFP/RIP1.DsRed2 [22] the rbGK.EGFP cassette by TRE-tight.mLeptin-IRES-mCherry. Next, we exchanged in pENTR.TRE-tight.mLeptin-IRES-mCherry/RIP1.DsRed2 the DsRed cDNA by the TetOFF-IRES-ZsGreen cassette obtained from pTetOFF-Dual (Green) (Clontech, Takara, Mountain View, CA, USA), thus generating pENTR.TRE-tight.mLeptin-IRES-mCherry/RIP1.TetOFF-IRES-ZsGreen. All constructs were verified by DNA sequence analysis and leptin expression was verified by Simple Western. The TRE-tight.mLeptin-IRES-mCherry/RIP1.TetOFF-IRES-ZsGreen cassette was transferred into the promoter-less adenovirus plasmid pAd/PL-DEST (Thermo Fisher Scientific, Waltham, MA, USA) by the Gateway technique. The ViraPower Adenoviral Expression System (Thermo Fisher Scientific, Waltham, MA, USA) was used to generate a replication-deficient adenovirus called vAd-RIP-leptin-OFF, which was used for transduction of cells.

### 4.4. Isolation of Pancreatic Islets

Islets were isolated from B6.BKS(D)-Lepr^db^/J (*db/db*) mice. Islets were prepared from mice by duct injection of collagenase (F. Hoffmann-La Roche, Basel, Switzerland) and were handpicked under a stereomicroscope MZ6 (Leica Microsystems, Wetzlar, Germany) after digestion. Thereafter, islets were cultured in RPMI-1640 medium (RPMI medium, Gibco, Thermo Fisher Scientific, Waltham, MA, USA), with a final concentration of 10% heat-inactivated fetal bovine serum, 2 mM glutamine, 100 U/mL penicillin, and 100 mg/mL streptomycin (all from Gibco, Thermo Fisher Scientific, Waltham, MA, USA) at 5% CO_2_ and 37 °C.

### 4.5. Islet Organoid Production

The islets were collected into 1.5 mL Eppendorf tubes (250 islets/tube), digested with accutase (Sigma-Aldrich, St. Louis, MO, USA) for 10 min at 37 °C and centrifuged at 500 rpm. The islet cells were transduced with 4 × 10^6^ plaque forming units/mL of the leptin encoding adenovirus in suspension culture dishes for 1h in RPMI medium at 5% CO_2_ and 37 °C and then washed two times with an excess of RPMI medium to get rid of the adenovirus. 2500 islet cells were seeded into each well of Nunclon Sphera 96U Bottom plates (Thermo Scientific, Leicestershire, UK). Islet organoids formed over a time of 5 days, after which the organoids were transferred to suspension culture dishes.

### 4.6. In Vitro Imaging of Intraocular Islet Organoids

An inverted laser scanning confocal microscope (TCS SP8, Leica Microsystems, Wetzlar, Germany) was used to image islet organoids as 3-dimensional stacks with a 3-µm step size. ZsGreen fluorescent protein was excited at 488 nm, and the fluorescence was detected at 505–535 nm, mCherry was excited at 561 nm, and fluorescence was detected at 580–650 nm. Backscatter signal (reflection) from the 561-nm excitation was collected at 555–565 nm.

### 4.7. Leptin Secretion from Islet Organoids In Vitro

Islet organoids were cultured in vitro in suspension culture dishes in RPMI medium. For glucose-stimulated leptin secretion measurements the organoids were placed into 4-well-plate (Nunc, Thermo Fisher Scientific, Waltham, MA, USA) suspension dishes containing RPMI medium with 3 mM glucose for 1.5 h. After that, the organoids were first placed into new wells containing medium with 3 mM glucose for 30 min and then later into new wells containing 16 mM glucose for 30 min. Then the organoids were returned into suspension culture dishes containing normal RPMI medium for continued culture. The medium from the 4-well-plate wells was collected into Eppendorf tubes, centrifuged for 1 min to pellet possible organoid fragments. The supernatants were collected into fresh tubes and kept at −20 °C until analysis.

### 4.8. Transplantation of Islet Organoids into the ACE

The islet organoids were transplanted into the ACE of three (first set of experiments) and seven (second set of experiments) *ob/ob* recipients, using a technique previously described in [23]. Briefly, under anesthesia, organoids were transplanted into the ACE with a glass cannula after generating a puncture in the cornea with a 27-gauge needle. Great care was taken to avoid bleeding and damage to the iris. Each mouse of the transplanted group received 100 organoids/eye. Mice were injected subcutaneous with Temgesic (0.1 mL/kg; RB Pharmaceuticals, Berkshire, UK) for postoperative analgesia. Three *ob/ob* mice (first set of experiments) and five *ob/ob* mice (second set of experiments) were used as controls and were not transplanted.

### 4.9. In Vivo Imaging of Intraocular Islet Organoid Grafts

Islet organoid grafts were imaged in vivo, beginning 9 weeks after transplantation. An upright laser scanning confocal microscope (TCS SP5, Leica Microsystems, Wetzlar, Germany), equipped with a long-distance, water-dipping objective (HXC-APO10x/0.30 NA, Leica Microsystems, Wetzlar, Germany) and a custom-built stereotaxic head holder, allowing positioning of the mouse eye containing the engrafted islets toward the objective, was used. Viscotears (Thea Nordic, Örebro, Sweden) was used as an immersion liquid between the eye and the objective, and isoflurane was used to anesthetize the mice during in vivo imaging. Grafts were imaged as 3-dimensional stacks with 3-µm step size. ZsGreen fluorescent protein was excited at 488 nm, and the fluorescence was detected at 505–535 nm. mCherry was excited at 561 nm, and fluorescence was detected at 580–650 nm. Backscatter signal (reflection) from the 561-nm excitation was collected at 555–565 nm. After imaging, the mice were allowed to recover from anesthesia. Additionally, beginning 6 weeks after transplantation, overview images of the grafts were obtained using a digital camera connected to a Leica M60 stereomicroscope (Leica Microsystems, Wetzlar, Germany) while the mice were under anesthesia.

### 4.10. Doxycycline Treatment of Animals

Three animals of the transplanted group were treated with doxycycline (Clontech, Takara, Mountain View, CA, USA) in order to stop leptin production from the islet organoid grafts. Sterile doxycycline hydrochloride, dissolved in PBS, was administered intraperitoneally 5 times over 10 days (50 µg/kg/mouse), starting on day 53 after stopping leptin treatment.

### 4.11. Body Weight and Fasting and Non-Fasting Blood Glucose

Body weight was determined before 9 am with full access to food. Fasting blood glucose was measured after 6 h denial of food. Non-fasting blood glucose was measured at 4 pm with full access to food.

### 4.12. Intraperitoneal Glucose Tolerance Test (ipGTT)

To determine glucose tolerance, blood glucose levels were measured in mice that were unfed for 6 h at basal state (0 min) and at 10, 30, 60, and 120 min after glucose injection (2 g/kg bodyweight intraperitoneally, dissolved in PBS). The results were depicted as the area under the curve (AUC) of the ipGTT. Glucose concentrations were measured with the Accu-Chek Aviva monitoring system (F. Hoffmann-La Roche, Basel, Switzerland). If blood glucose concentrations exceeded the detection limit of the glucose meter, the limit value of 33.3 mM was used as value for the determination of the AUC of the ipGTT.

### 4.13. Plasma and Aqueous Humor Samples

Blood samples were taken in the mornings from non-fasted animals and collected into Microvette CB300 EDTA/PK100 tubes (Sarstedt, Nürnbrecht, Germany), centrifuged to gain blood plasma, and preserved at −20 °C until use. Aqueous humor samples were obtained at the end of the experiment and kept at −20 °C until use.

### 4.14. Insulin and C-Peptide Measurements

Ultrasensitive mouse ELISA kits (Crystal Chem, Elk Grove Village, IL, USA) were used to analyze insulin and C-peptide levels in the plasma.

### 4.15. Leptin Measurements

Leptin was measured in cell culture medium, blood plasma, and aqueous humor samples using the Mouse/Rat Leptin Quantikine ELISA Kit (R&D Systems, Minneapolis, MN, USA).

### 4.16. Tissue Extraction and Sectioning

Mice were anesthetized with isoflurane and sacrificed by cervical dislocation. Eyes were extracted and fixed with 4% paraformaldehyde for 1 week. Before cryopreservation, the eyes were processed with a sucrose gradient (10–30% (wt/vol) sucrose in PBS containing 0.01% (wt/vol) sodium azide and 0.02% (wt/vol) bacitracin, embedded in OCT-Compound (Tissue-Tek, Sakura Finetek, Torrance, CA, USA), frozen on dry ice, and preserved at −80 °C until use. Then, 20 µm thick cryosections of the anterior part of the eye were collected on SuperFrost Plus microscope slides (VWR International, Radnor, PA, USA) and kept at −20 °C until use.

### 4.17. Immunofluorescence in Eye Sections

For immunostaining, eye sections were equilibrated to room temperature, washed, blocked, and incubated with the primary antibodies, goat anti m-leptin (R&D Systems, Minneapolis, MN, USA) and rabbit anti-C-peptide (Cell Signaling, Danvers, MA, USA) in the presence of 0.1% Triton X-100 and 10% serum. After washing, secondary antibodies, anti-goat Alexa633, and anti-rabbit Alexa594, respectively (Thermo Fisher Scientific, Waltham, MA, USA), were applied, and mounting with medium containing DAPI for nuclear counterstaining (Thermo Fisher Scientific, Waltham, MA, USA) was performed after repeated washing. Imaging was performed using a confocal laser scanning microscope (Leica TCS SP8, Leica Microsystems, Wetzlar, Germany) with the following settings: DAPI excitation 405 nm, detection 450–470 nm; ZsGreen excitation 488 nm, detection 500–525 nm; mCherry excitation 548 nm, detection 560–580 nm; Alexa 594 (C-peptide staining) excitation 594 nm, detection 600–620 nm and Alexa 633 (leptin staining) excitation 633 nm, detection 640–680 nm. To avoid spectral overlap imaging was performed using in-between-frames sequential imaging.

### 4.18. Statistics

The values are expressed as means ± SEM. A 2-sided, unpaired t test was used to determine statistical significance among different treatment groups. Statistical significance was determined as follows: * *p* < 0.05, ** *p* < 0.01 and *** *p* < 0.001. Origin 2015 64-bit (OriginLab, Northampton, MA, USA) and Excel (Microsoft, Redmond, WA, USA) were used for statistical analyses.

## 5. Conclusions

Our data provide a proof-of-concept that genetically engineered islet organoids can serve as production sites for blood-born proteins/peptides.
